# Diagnostic Performance of Three ELISAs for Detection of Antibodies against SARS-CoV-2 in Human Samples

**DOI:** 10.1155/2022/7754329

**Published:** 2022-08-16

**Authors:** Cássio Meira, Dahara Silva, Ivanilson Santos, Breno Barreto, Vinícius Rocha, Emanuelle Santos, Bruna dos Reis, Afrânio Evangelista, Ricardo Ribeiro dos Santos, Bruna Machado, Guilherme Ribeiro, Roberto Badaró, Milena Soares

**Affiliations:** ^1^SENAI Institute of Innovation in Health Advanced Systems (CIMATEC ISI SAS), University Center SENAI/CIMATEC, Salvador, Brazil; ^2^Gonçalo Moniz Institute Oswaldo Cruz Foundation (IGM-FIOCRUZ/BA), Salvador, Brazil; ^3^School of Medicine, Federal University of Bahia (UFBA), Salvador, Brazil

## Abstract

Severe acute respiratory syndrome coronavirus 2 (SARS-CoV-2) infection that causes coronavirus disease 2019 (COVID-19) is a disease with a high rate of transmission. Serological tests are important to perform surveys and to determine the immunological status of the population. Based on this, we evaluated three enzyme-linked immunoassays (ELISAs) using different antigens from SARS-CoV-2 in a cohort of 161 patients. The performance of the ELISA developed for immunoglobulin G (IgG) measurement against SARS-CoV-2 was evaluated based on sensitivity, specificity, and accuracy. We found specificities of 0.98, 0.98, and 0.99 and sensitivities of 0.99, 0.91, and 0.87 for the nucleocapsid (N) protein, spike protein, and receptor binding domain (RBD) fraction, respectively. The accuracy assessment indicated the N protein (accuracy = 0.98) as the antigen most likely to give a correct diagnosis. Overall, the antibody responses were present for all three proteins in subjects with confirmed SARS-CoV-2 infections, showing a similar pattern of antibody production for different antigens. In summary, these highly sensitive and specific ELISAs, with a more competitive price, appear to be a valid approach for the serodiagnosis of COVID-19.

## 1. Introduction

In December 2019, in Wuhan, China, there was an outbreak of pneumonia cases of unknown etiology [1]. In January 2020, the Chinese government isolated the etiologic agent and described it as a new coronavirus associated with a severe acute respiratory syndrome coronavirus 2 (SARS-CoV-2), known as coronavirus disease 2019 (COVID-19) [2, 3]. The World Health Organization (WHO), in March 2020, declared COVID-19, as a pandemic and, according to their data, in April 2022, the world was facing more than five hundred million confirmed cases worldwide and the death toll surpassed six million people [4].

SARS-CoV-2 is an enveloped single-stranded RNA virus and has four structural proteins: the nucleocapsid protein (N), responsible for packaging the genomic RNA and together constitutes the nucleocapsid; the surface spike protein (S), composed of S1 subunit and S2 subunit, allows the attachment and binding with the host cell receptors (S1) and fusion of the cell membrane and viral membrane (S2); the envelope protein (E) and the membrane protein (M), together mediate virion budding [5–9]. The surface S1 subunit is organized into domains, among them, the receptor binding domain (RBD) is involved in host cell penetration by binding to the angiotensin-converting enzyme 2 (ACE2), being considered a key protein for SARS-CoV-2 infection [10, 12]. In addition to mediating the entry of the virus into host cells, RBD is one of the main targets of human antibodies against COVID-19 and has shown to be a promising antigen for the detection of specific antibodies [12–15].

Due to the high transmission rate of SARS-CoV-2 and the absence of effective treatment, diagnostic tools have remained crucial to identify infected individuals quickly and avoid high transmissibility rates [16]. The gold standard test for the diagnosis of SARS-CoV-2 infection is the reverse transcription polymerase chain reaction (RT-PCR) test, which detects the virus nucleic acid [17]. Despite being a highly sensitive method that can successfully detect SARS-CoV-2 infection during the acute phase of infection, false-negative cases have been reported related to factors such as viral load, sample collection, RNA extraction, enzyme inhibitors, and the RT-PCR method [16, 18]. Therefore, in relation to epidemiological investigations, detection of virus nucleic acid would not be useful for diagnosis if these infected individuals recovered and no longer shed the virus [19, 20].

As an attractive alternative, serological tests, such as enzyme immunoassay (ELISA) for detection of immunoglobulin A (IgA), G (IgG), and M (IgM) antibodies, have been widely used to aid in the diagnosis of SARS-CoV-2 infection and, recent studies have shown a positive correlation between high titers of IgG antibodies with neutralizing antibodies in COVID-19 [21]. In addition, serological assays are critical to understanding the epidemiology of SARS-CoV-2 infection, postvaccination monitoring and identification of recovered COVID-19 patients for convalescent plasma therapy [21, 22]. N and S proteins, due to their higher immunogenic properties, are the main proteins and are used as antigens in serological assays for the diagnosis of SARS-CoV-2 infection [22–25].

Seroconversion for SARS-CoV-2 is estimated to occur 7–14 days after the onset of symptoms, when the sensitivity of the PCR decreases, making the use of RT-PCR and ELISA, complementary techniques to increase the sensitivity of the diagnosis of SARS-CoV-2 infection [26]. Previous investigations already show that the combined use of RT-PCR associated with serological methods shows greater sensitivity when compared to isolated RT-PCR, making this strategy attractive to limit the virus spread [19]. ELISA also can provide epidemiological information regarding the number of affected individuals in a population, guide control measures taken by governments, and be useful to evaluate the efficacy of vaccines against SARS-CoV-2 [27, 28].

Based on the importance of having a robust method for the massive serological detection of previous infections in the community, especially for postvaccination monitoring, we developed and validated three ELISA assays using different antigens from SARS-CoV-2. Here, we describe the performance of these assays, which are based on the N, S, and the receptor binding domain (RBD) of the S protein. Also, we discussed the clinical implications related to the use of these antigens to diagnose COVID-19.

## 2. Materials and Methods

### 2.1. Antigen Production

The spike glycoprotein was expressed in human embryonic kidney (HEK) cell line 293T and purified from the supernatant of the cells as previously described with certain modifications [12]. Briefly, HEK 293T cells were cultivated in 175 cm^2^ cell culture flasks and transfected with the pCAAG-spike vector (kindly provided by Florian Krammer from the Department of Microbiology, Icahn School of Medicine at Mount Sinai, New York, NY), using the ExpiFectamine293 reagent (Thermo Fisher, Waltham, MA).

After 48 hours of transfection, the supernatant was collected, and the recombinant his-tagged proteins were purified through Ni-Sepharose columns (Cytiva, Chicago, IL). The recombinant protein was characterized by western blotting using anti-spike antibody (MyBiosource, San Diego, CA) diluted 1 : 500 (Figure S1). A secondary goat anti-mouse horseradish peroxidase-conjugated antibody was used at 1 : 5000 (Santa Cruz Biotechnology, Dallas, TX). The samples were detected using an Opti-4CN Substrate Kit (Bio-Rad, Hercules, CA). The nucleocapsid protein (N) and RBD fraction from the spike protein were purchased from MyBioSource (San Diego, CA).

### 2.2. Samples

This study was approved by the local ethics committee (approval number: 4.334.505). To assess the specificity, samples from 92 subjects were selected. This cohort contained 69 serum samples collected before October 2019, including 15 convalescent samples from patients with RT-PCR-confirmed chikungunya virus infection and 15 convalescent samples from patients with RT-PCR-confirmed dengue. The cohort also included serum samples collected between February and March 2020 from 23 patients without previous history of COVID-19 symptoms, without recent travel history, without contact with positive people for COVID-19 and with RT-qPCR negative for SARS-CoV-2. In addition, to determine the sensitivity of the assay, samples from 69 patients with a positive diagnosis for COVID-19 by RT-qPCR were collected between 15 to 30 days after positive molecular diagnosis. The serum samples were separated after centrifugation at 3000 rpm for 10 min. All sera samples were stored at −20°C before use.

### 2.3. Antibody Detection

**Figure 1:**
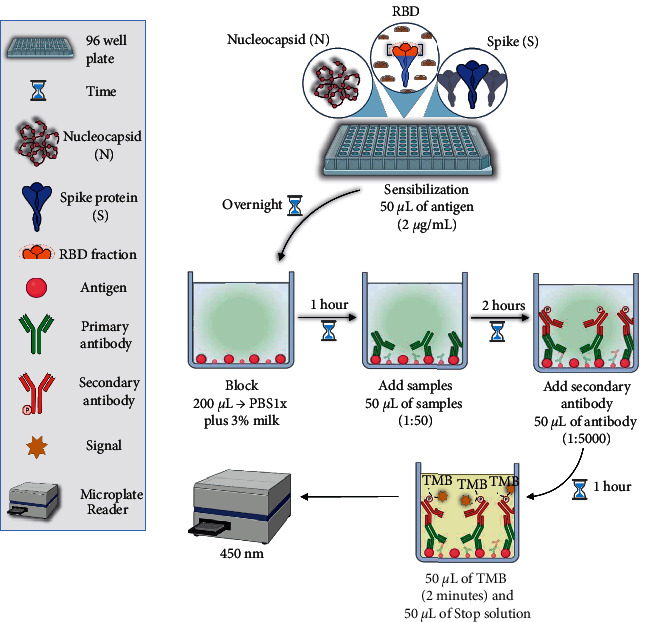
Flowchart of the in-house ELISAs developed. ELISA plates were coated with 50 *μ*L of the different antigens (N, S, or RBD fraction) at 2 *µ*g/mL. After 16 hours, the plates were washed with PBS and blocked with a solution of PBS containing 3% non-fat milk and 0.05% Tween 20 for 1 hour. Next, the serum samples were diluted 1 : 50 in PBS containing 1% nonfat milk and 0.05% Tween 20 and added to plates coated with SARS-CoV-2 antigens. Following 2 hours of incubation, the plates were washed and incubated with horseradish peroxidase-labeled anti-human IgG secondary antibody (1 : 5000 dilution in PBS containing 1% nonfat milk and 0.05% Tween 20). The plates were then washed following 60 min incubation at 37°C, and 3,3′,5,5′-tetramethylbenzidine substrate was added. Two min later, stop buffer was added, and the absorbance values were measured at 450 nm wavelength using a microplate reader.

IgG antibodies against SARS-CoV-2 were measured by ELISA, as previously described [12] (Figure 1). In brief, ELISA plates were coated with 50 *μ*L of the different antigens (N, S, or RBD fraction) at 2 *µ*g/mL, in phosphate-buffered saline (PBS), at pH 7.2. The plates were incubated at 4°C overnight. After 16 hours, the plates were washed three times with PBS containing 0.05% Tween 20 (wash solution) and blocked with a solution of PBS containing 3% nonfat milk and 0.05% Tween 20 (Sigma-Aldrich, St. Louis, MO) for 1 hour. Next, the serum samples were diluted 1 : 50 in PBS containing 1% nonfat milk and 0.05% Tween 20 and added to plates coated with SARS-CoV-2 antigens.

Following 2 hours of incubation at 37°C, the plates were washed three times and incubated with horseradish peroxidase-labeled anti-human IgG secondary antibody (1 : 5000 dilution in PBS containing 1% nonfat milk and 0.05% Tween 20 (Bio-Rad, Hercules, CA). The plates were then washed following 60 min incubation at 37°C, and 3,3′,5,5′-tetramethylbenzidine substrate (Scienco, Lages, SC, Brazil) was added. Two min later, stop buffer (phosphoric acid 1 M) was added, and the absorbance values were measured at 450 nm wavelength using a microplate reader. The results were reported as the optical density (OD). The cutoff values were determined by calculating the mean absorbance at 450 nm (A450) of the negative sera plus two-fold of the standard deviation values.

### 2.4. Statistical Analysis

The performance of the ELISA assays developed for IgG measurement against SARS-CoV-2 was evaluated based on sensitivity, specificity, and accuracy. The analyses were performed using the unpaired Student's *t*-test and Pearson's correlation. All analyses were performed using GraphPad Prism version 5.01 (GraphPad Software, San Diego, CA). The experiments were performed three times in duplicate.

## 3. Results

**Figure 2:**
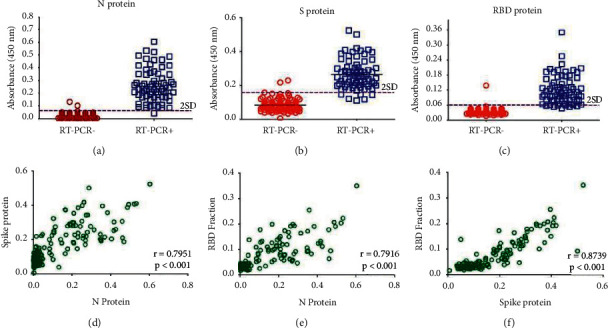
ELISA performed with different severe acute respiratory syndrome (SARS-CoV-2) proteins used as antigens. (a–c) IgG anti-SARS-CoV-2 detected by ELISA using N (a), S (b), and RBD (c) antigens. The cutoff was set as the mean plus two standard deviations of the healthy control samples. The correlations of the ELISA values between N and S (d), N and RBD (e), and S and RBD (f) antibody values. The Pearson correlation coefficient (r) was used to measure the strength of the correlation between the ELISA results performed with different antigens. The results are from one experiment of the three experiments performed.

Initially, we used a collection of 92 control samples (69 samples obtained before the COVID-19 pandemic and 23 samples from healthy individuals with negative RT-qPCR for SARS-CoV-2) to calculate the cutoff values for each of the proteins used as an antigen. As shown in Figures 2(a)–2(c), the cutoff values found were 0.059, 0.16, and 0.06 for the N protein, Spike protein, and RBD fraction, respectively.

Using these cutoff values, we found a specificity of 0.98, 0.98, and, 0.99 for the N protein, Spike protein, and RBD fraction, respectively, indicating a high specificity for all three antigens. Interestingly, convalescent samples from patients with RT-PCR confirmed for chikungunya and dengue virus infection did not show cross-reactivity against SARS-CoV-2 antigens. Next, we used the above cutoff values to screen a collection of positive samples (69 samples obtained from patients with COVID-19 confirmed by RT-PCR), and found sensitivities of 0.99, 0.91, and, 0.87 for the N protein, Spike protein, and RBD fraction, respectively (Table 1). Therefore, the best performance of the ELISA test was found with the use of the N protein. The accuracy assessment indicated the N protein (accuracy = 0.98) as the antigen most likely to produce a correct diagnosis (Table 1).

**Figure 3:**
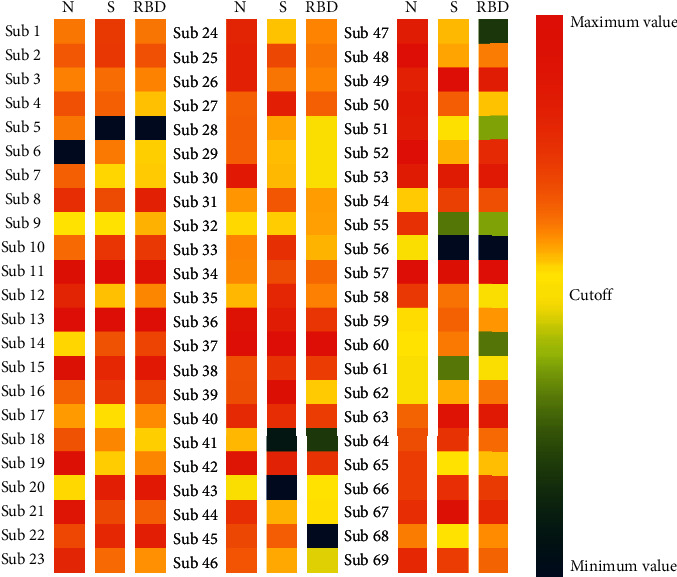
Heatmap showing the average reactivities to the three antigens in individual patients. The heatmap was constructed using only the 69 patients with a positive diagnosis for COVID-19 by RT-qPCR, and the samples were collected between 15 to 30 days after a positive molecular diagnosis. The absorbance values found for each protein were normalized based on the cutoff point found for each protein (N, S, and RBD fraction). The color gradient bar represents lower reactivity against antigens in dark blue and higher reactivity against antigens in dark red.

Last, we found a strong correlation between the S protein and RBD fraction (*r* = 0.8739; *p* < 0.001) (Figure 2(d)), although the correlations between the N protein and S protein (*r* = 0.7951; *p* < 0.001) and the N protein and RBD fraction (0.7916; *p* < 0.001) were also significant (Figures 2(e) and 2(f)). Overall, the antibody responses were present for all three proteins in the subjects with confirmed SARS-CoV-2 infection, showing a similar pattern of antibody production for different antigens, especially between S protein and RBD fraction (Figure 3).

## 4. Discussion

The immune response against SARS-CoV-2, particularly IgG antibody production has been shown to be essential for the epidemiological monitoring of SARS-CoV-2 infection and validation of new vaccines. IgG production can also be also associated with clearance of the virus infection and prevent future reinfections when neutralizing IgG antibodies are produced [27–29]. In addition, IgG detection is pivotal in the serodiagnosis of COVID-19. Our study demonstrated that in-house ELISAs with three different SARS-CoV-2 antigens, separately, as plate sensitizers (N, S, and, RBD fraction), were a useful tool for COVID-19 serological diagnostics with specificities and sensitivities better than commercially available immunoassays with more competitive prices [30].

ELISA tests for IgG detection performed with the N protein, well-known for its high immunogenicity and intracellular accumulation before packing of the virus, showed the best performance with the highest accuracy [31]. Noteworthy, previous studies regarding immunologic responses against the coronavirus subfamily indicated that the IgG antibody response against the N protein was more prominent than against the S protein [32, 33]. In addition, previous studies with SARS-CoV-1 demonstrate that the serum durability of antibodies against the N protein is greater than antibodies against the S protein, making its use attractive for tracking viral infections for longer periods [34, 35]. Another advantage of using the N protein as an antigen is the possibility of differentiating infected and noninfected vaccinated persons producing anti-S-protein antibodies. Although our data are in accordance with reports in the literature, a limitation of our work was the use of a single antigen, not taking into account the possible variability that may exist in the protein production and purification procedure [34].

The N antigen also presents high immunogenicity against coronavirus from elk and the infectious bronchitis virus, an avian coronavirus [36, 37]. Despite the high immunogenicity profile of N protein, previous reports showed no cross-reactivity of SARS-CoV-2 N protein with human plasma positive IgG antibodies against other human coronaviruses such as NL63, 229E, OC43, and HKU1 [19, 33]. Yet, a strong cross-reactivity was found in human plasma with positive IgG antibodies against SARS-CoV-1 [19].

In addition, we found a strong correlation between the ELISA performed with the N protein versus the S protein or RBD fraction, similar to previously reported [38]. Our data also reinforce that combined detection of N and S protein or N and RBD fraction improves the IgG serological detection. Indeed, this combination increases the accuracy of antibody detection [19]. The strongest correlation found was observed in the S protein versus RBD fraction, which was expected since the RBD corresponds to the S1 subunit of the S protein. However, it is important to note that the RBD fraction represents only a small part of the spike protein (237 amino acids in RBD as compared to 1273 amino acids in the S protein), so the production of IgG antibodies against RBD may not represent the production of IgG antibodies against the spike protein, which was reinforced by our results [19].

The S protein is essential for binding to the host receptor angiotensin 2-converting enzyme (ACE2) [10, 39–41]. The S protein and the RBD fraction of Middle East respiratory syndrome coronavirus (MERS-CoV) have been used for the development of treatments, such as neutralizing anti-MERS-CoV, since they inhibit infection by blocking the virus from binding to the cell receptor or fusing with the cell membrane [42].

Therefore, the generation and maintenance of neutralizing antibodies against SARS-CoV-2, typically target the S protein and RBD fraction, which plays an important role in the resistance to infection by the host, blocking the interaction between the virus and the recipient host [43–45]. An assay, such as the one standardized in this study, may be useful in the postvaccine months/years to help the public health system to determine the reality regarding anti-S antibody titers.

In summary, ELISA-based antibody detection appears to be a valid approach to the serodiagnosis of COVID-19. ELISA is simple, fast, cheap, and safe and requires a low amount of serum to be performed. All antigens (N protein, S protein, and RBD fraction) tested showed a satisfactory performance, with prominence of the N protein antigen, for the detection of specific IgG antibodies against the SARS-CoV-2 virus. The technique is not limited to diagnostic use. This ELISA method also allows for gathering epidemiological data to estimate the number of individuals previously infected, which can guide preventive measures used by governments to assess the effectiveness of the SARS-CoV-2 vaccine. More importantly, the test will allow monitoring of IgG levels in the postvaccination era and comparing the different immunizers used worldwide.

## Figures and Tables

**Table 1:** The overall diagnostic performance of enzyme-linked immunoassay (ELISA) performed with the N protein, S protein, and receptor binding domain (RBD) fraction as antigens.

Parameters	N protein	S protein	RBD
Sensitivity	0.99 (0.92–1.0)	0.91 (0.82–0.96)	0.87 (0.77–0.94)
Specificity	0.98 (0.92–1.0)	0.98 (0.92–1.0)	0.99 (0.94–1.0)
Accuracy	0.98 (0.95–1.0)	0.95 (0.90–0.98)	0.94 (0.89–0.97)

Values and the respective 95% confidence interval are in parentheses. Sensitivity = (True positive/(True positive + False negative)); Specificity = (True negative/(True negative + False positive)); Accuracy = ((True negative + True positive)/Total tests).

## Data Availability

The data that support the findings of this study are available from the corresponding author upon reasonable request.

## Supplementary Materials

Supplementary Materials Figure S1: a western blot was performed using the recombinant spike protein.
