# Supplementary material for: Diagnostic Performance of Three ELISAs for Detection of Antibodies against SARS-CoV-2 in Human Samples
Source: ScientificWorldJournal. 2022 Aug 16;2022:7754329. doi: 10.1155/2022/7754329 (PMC9398874; doi:10.1155/2022/7754329)
Supplement: Supplementary Materials — Figure S1: a western blot was performed using the recombinant spike protein. [file 7754329.f1.docx]

**Suplementary files**


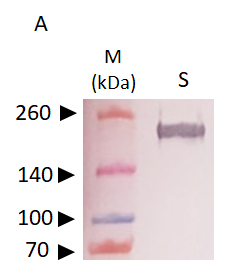


**Figure S1**. A western blot was performed using the recombinant spike protein. The sample was resolved in sodium dodecyl sulphate–polyacrylamide gel electrophoresis (SDS-PAGE) and transferred to the nitrocellulose membrane. The membrane was blocked in Tris buffered solution containing 5% (w/v) non-fat milk. The protein was specifically detected using an anti-spike antibody. A secondary anti-mouse horseradish peroxidase-conjugated antibody was used.
